# The Use of Calcium Sulphate beads in Periprosthetic Joint Infection, a systematic review

**DOI:** 10.7150/jbji.41743

**Published:** 2020-02-10

**Authors:** Abdulbaset Abosala, Mohammed Ali

**Affiliations:** 1 Orthopaedic Consultant in Trauma and Orthopaedics, Manchester University Hospital NHS Foundation Trust, UK; 2 Speciality registrar in Trauma and Orthopaedics, Raigmore Hospital, Scotland, UK

**Keywords:** calcium sulphate beads, periprosthetic joint infection, hip revision, knee revision

## Abstract

**Purpose**: To assess the use of calcium sulphate (CS) beads in the management of knee and hip periprosthetic joint infections (PJI) in terms of outcomes, complications and re-infection rates.

**Methods**: A search of NICE healthcare database advanced search (HDAS) was conducted from its year of inception to October 2019 with the keywords: “Calcium Sulphate Beads” or “Calcium Sulfate Beads” or “Antibiotics beads” or “Stimulan” and “Arthroplasty” or “Hip Replacement” or “Knee Replacement” and “Periprothetic joint infection” or “Debridement, Antibiotics and implant retention” or “Revision”. A quality assessment was performed using the NIH study Quality Assessment Tool for case series.

**Results**: Out of relevant 74 articles, 5 articles met the inclusion criteria. Variable outcomes and success rates have been reported in most of the patients. A small number of wound discharges and heterotrophic ossification (HO) were reported, which are occasionally symptomatic. Hypercalcemia is identified as a potential risk with the use of CS beads especially with doses over 40 cc per operation. The influence of CS beads on reinfection rate is reported in 4 out of 5 articles. Due to the case-mix and heterogenicity of the patients involved and the causative microorganism reported as well as varied procedures are undertaken including one and two stages revision and Debridement, Antibiotics and implant retention (DAIR), the influence of CS beads varied from high success to poor outcome. The poor outcome is higher if the primary procedure for the management of hip and knee PJI is DAIR rather than full revision.

**Conclusions**: The use of CS beads in the treatment of PJI is a useful technique in delivering high doses of antibiotics locally. A favourable outcome is reported when antibiotics loaded CS is used as an adjuvant to revision procedure for PJI in hip and knee arthroplasty compared to its use as an adjuvant to DAIR procedure. There has been an increase in complications when higher volumes of beads are used, especially in subcutaneous structures and in high-risk patients. Another possible theoretical and unreported complication of CS beads is accelerating the wear rate in the artificial joint due to the possibility of causing abrasion to the bearing surfaces. The current evidence is not enough to indicate the superiority of antibiotic-loaded CS beads as an adjuvant for the treatment of PJI in Hip and Knee arthroplasty.

## Introduction

PJI is a devastating complication for patients and results in greatly increased costs of care for both healthcare providers and patients. PJI occurs in around 1-2% of primary total hip and knee arthroplasties 1, 2, 3. Coagulase-negative Staphylococci are diagnosed in 30-41% and *Staphylococcus aureus* in 12-47%. Streptococci and Enterococci are diagnosed in around 10% while gram-negative bacteria such as *Escherichia coli* are less than 5% 4, 5, 6. A DAIR procedure is usually utilised for acute infections without complicating factors such as significant comorbidity or loosening of the prosthesis. However, DAIR has a variable success rate in eradicating PJI depends on several factors including the duration of infection, the host immunity, the virulence of the causative microorganism and the technique employed. Due to the poor vascularity and the formation of the biofilm infected artificial joints are often unresponsive to systemic antibiotic treatment 7. Once the biofilm is formed more aggressive surgical techniques are employed and these will include a single-stage or two-stage revision procedures. In addition to adding systematically targeted antibiotics, surgeons started to use local antibiotics by implanting antibiotic-loaded carrier material, to deliver antibiotics at high concentration directly at the site of the infection. Initially, polymethylmethacrylate (PMMA) cement was used as a carrier however, it has some undesirable characteristics 8. PMMA beads require subsequent removal and may develop biofilm on their surface if left in situ for long periods 9. Some authors have shown a relatively short period of antibiotic release with a decrease in local concentrations to 10% of the initial levels within 24 hours 10. Currently, CS beads are used as an alternative void filler to PMMA in the presence of infection, non-union or bone loss 11. As it is absorbed, CS releases 100% of its antibiotic load, resulting in superior elution characteristics and higher sustained antibiotic concentrations over a period of several weeks 12. This results in concentrations of antibiotic locally that can be many times higher than the minimum inhibitory concentration for the relevant pathogen, while also ensuring that systemic levels and associated toxicity remain low 13,14. Other authors recommended tailoring the dose of Aminoglycosides like Tobramycin to the patient's renal function rather than weight in cases of severely impaired renal function 15.

The use of CS in orthopaedics has therefore been increasing, both as a bone void filler and as a delivery agent for antibiotics in arthroplasty, chronic osteomyelitis, open fractures and combat injuries 11. As this practice has increased, so has the understanding of the associated benefits and complications, which include transient hypercalcaemia, wound drainage and HO. This review aims to assess the outcomes of using antibiotics loaded CS beads in the management of hip and knee PJI focusing on reinfection rates and complications.

## Methods

The Preferred Reporting Items for Systematic Review and Meta-Analysis (PRISMA) methodology guidance was employed 16. We utilised the Healthcare Databases Advanced Search (HDAS) tool to search the MEDLINE, EMBASE, CINAHL, EMCARE and PUBMED databases. The search was conducted from its year of inception to October 2019 with the keywords: “Calcium Sulphate Beads” or “Calcium Sulfate Beads” “Antibiotics beads” or “Stimulan” and “Arthroplasty” or “Hip Replacement” or “Knee Replacement” and “Periprosthetic joint infection” or “Debridement, Antibiotics and implant retention” or “Revision”. We only included articles written in English language. Abstracts from the search were reviewed for relevant articles by two authors (MA and AA). All references listed in the relevant articles were also reviewed for any other papers not found in the initial search. Studies were included if they discuss the use of CS beads in hip and knee arthroplasty surgery as an adjunct in revision and DAIR procedures for the management of PJI. Due to the small number of studies, we did not set a minimum follow up period or minimum patients' number. Case reports, reviews, conference abstracts, studies on animals and technical notes were excluded. Papers which included the use of CS beads in the management of osteomyelitis or bone non-union were also excluded. Once relevant papers were identified, data was extracted using a standardised form for each of the following: Author, year of publication, study design, level of evidence, number of joints, inclusion criteria, type of joints, beads characteristics, antibiotics, follow-ups and complications specific to CS beads including wound discharge, HO and hypercalcemia, in addition to the reinfection rate following intervention. Due to the heterogeneity of the included data, a meta-analysis could not be conducted and therefore all data were reported descriptively. We used the NIH study Quality Assessment Tool for case series to assess the quality of the included papers (Table 1).

## Results

### Search results

**Figure 1:**
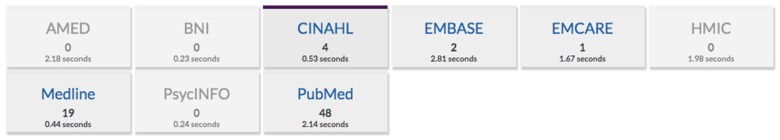
The results of the literature search.

The search threads used resulted in 74 articles (Figure 1).

**Figure 2:**
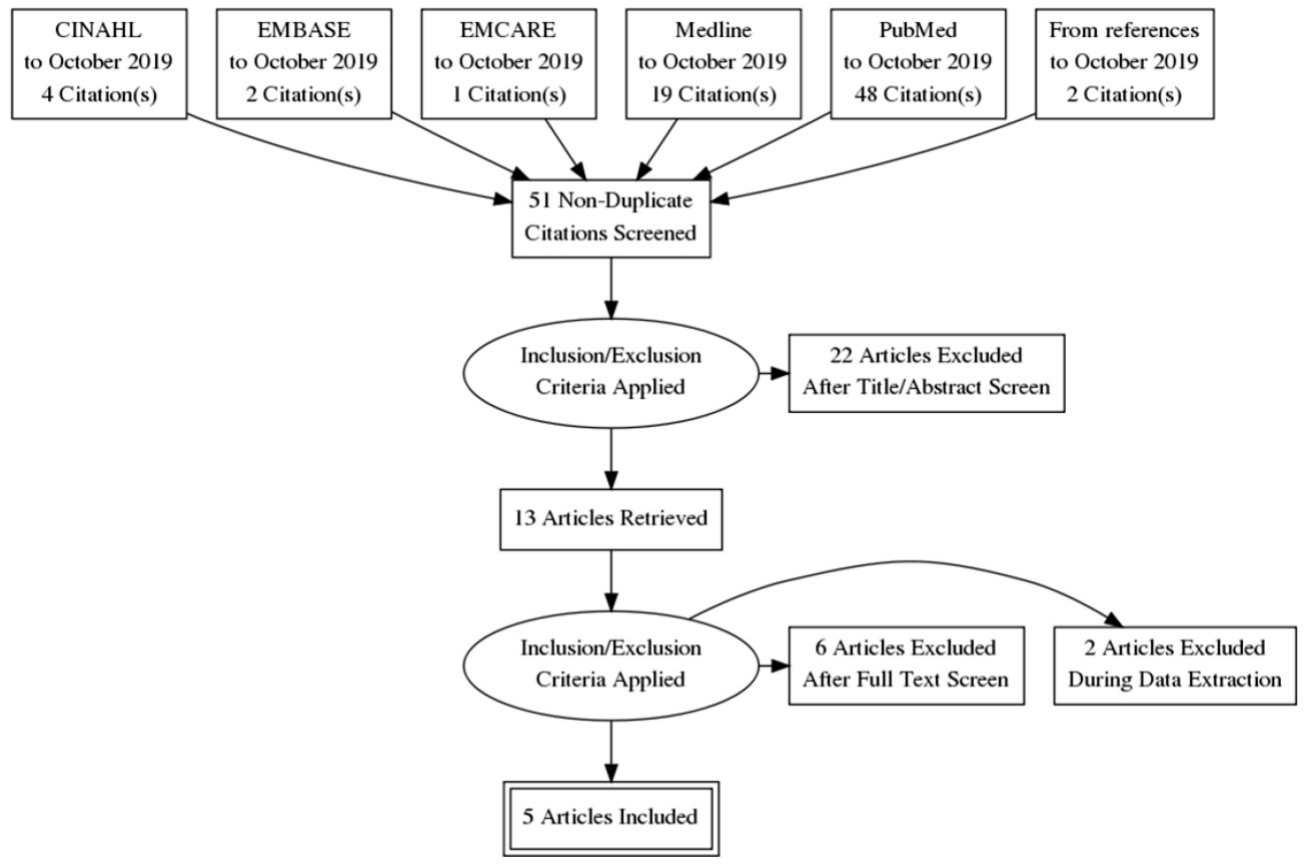
The PRISMA flow diagram.

Screening the references, we identified two more relevant articles. Eventually, out of the 76 papers, 5 papers were found to meet the inclusion criteria (Figure 2).

### Summary of studies

5 articles 17-21 met the inclusion criteria (Table 2). The 5 studies were published between 2013 and 2018. These studies included 1109 patients. All patients either had a primary or a revision of a total knee replacement (TKR) or a total hip replacement (THR) (Table 1). The mean follow-up varied from 3 months to 35 months. These series included 657 knees and 452 hips. The male: female ratio has been reported in 3 papers. Using the United States National Institute of Health (NIH) National Heart, Lung and Blood Institute (NHLBI) Quality Assessment Tool for case series studies 22, we rated 3 studies (Kallala 2015^18^, Kallala 2018 21 and Flierl 19 et al.) at a “good” quality rating and 2 studies (Lum (20)and McPherson 17 et al.) at a “fair” quality rating (Table 1). All papers included cases of knee and hip prosthetic infections. McPherson et al. 17 included aseptic revision in 124 joints out of 250. Kallala 2018 21 included 387 joints with PJI and the remaining 368 joints were other types of revision such as aseptic loosening, instability, periprosthetic fracture, metal allergy, implant failure and revisions for pain and stiffness. Lum et al. 20 used CS beads as prophylaxis in high-risk patients undergoing primary or revision arthroplasty as well as cases of PJI. Kallala 2015 18 and Flierl 19 et al. only included infected hip and knee arthroplasty (Table 2&4).

### Outcome measures

Different outcome measures were reported in these papers 17-21. Flierl et al. 19 measured the recurrence of infection using the Musculoskeletal Infection Society (MSIS) criteria while Lum et al. 20 reported wound complications such as persistent wound drainage, purulent exudate and local tissue irritation. Lum et al. 20 assessed patients for systemic toxicity, HO and the need for further surgery. Their secondary outcomes included reoperation and reinfection rates. McPherson et al. 17 reported post-operative reinfections and complications as primary outcomes. Kallala 2015^18^ paper focused on the reinfection rate and the complication of CS beads, however, Kallala 2018^21^ paper did not report any clinical or patient-reported outcome measures as these were not the focus of the study. They reported the incidence of persistent wound drainage, hypercalcaemia and HO (Table 2).

### Calcium sulphate beads Preparation

All authors in these papers used Stimulan from Biocomposites either from Keele in the UK or Wilmington in North Carolina, United States. The kit includes 10 cc (20 g) of calcium sulphate hemihydrate powder, a pre-mixing solution bulb, pellet mould and spatula. The mould produces three sizes of bead (3, 4.8 and 6 mm in diameter). One gram of vancomycin powder is mixed with each 10 cc of calcium sulphate in the mixing bowl and 240 mg of liquid tobramycin (40 mg/ml) is added. The ingredients were mixed for 30 seconds until “doughy” and the resulting paste was applied to the moulds using the spatula and allowed to set for 10 to 15 minutes in a typical theatre temperature of 16°c to 17°c. Kallala 2015^18^ reported using Gentamycin instead of Tobramycin and only allowed one to two minutes for the setting. Lum et al. 20 added Cefazolin to Vancomycin and Tobramycin. Kallala 2018 21 reported adding 50 mg of amphotericin B in patients with fungal infection (Table 3).

### Implantation Technique

The authors implanted the beads around the infected joint and deep tissues and avoided placing beads in subcutaneous layers (Table 3).

### Resorption time

The radiologically confirmed resorption time of calcium sulphate beads ranged between 3 to 12 weeks as reported by Kallala 2015 18, Lum 20 and McPherson et al. 17 (Table 4).

### Hypercalcaemia

Kallala 2015^18^ and Kallala 2018^21^ were the only to report hypercalcaemia (Table 3). Hypercalcaemia was reported in 44 patients, out of 770 (5.7%). The mean level was 11.7 mg/dl (10.8 to 14.9); the levels returned to normal at a maximum of five days postoperatively. Two patients from Kallala's 2018 21 series developed symptoms and were treated with one intravenous dose of bisphosphonate and 0.9% saline. One patient in Kallala's 2015 paper 18 developed confusion and lethargy and raised serum calcium level to 3.54 mmol/L (normal range 2.2 to 2.6) and required treatment in intensive care. Kallala 2018 21 noticed an increased risk of hypercalcemia with the use of a higher volume of CS beads. They recommended a maximum dose of 40 cc per operation and increased to 80 cc if it is placed within the medulla of the bone.

### Wound discharge

Flierl et al. 19 did not report the incidence of postoperative wound discharge, hence out of 1076 joints, persistent wound discharge was reported in 41 patients (3.8%) (Table 4). Kallala 2015 18 has no incidence of wound discharge. 3.2% in McPherson et al. series 17 developed wound discharge and the majority was found in medically compromised hosts of MSIS grade B and C. McPherson et al. 17 noted that a volume of CS beads of 30 cc or more is associated with persistent wound drainage. Lum et al. 20 reported one case of a medically compromised patient who developed a persistent wound discharge following the use of CS beads. This patient did not require any surgical intervention and the discharge stopped spontaneously. Kallala 2018 21 reported 32 patients with wound discharge. 23 of them underwent a washout procedure either because they had a persistent wound discharge for more than five days or the discharge was sanguineous.

### Heterotrophic ossifications

The authors used Harwin and Brooker classifications to describe the HO. Flierl et al. 19 did not report the incidence of HO, hence out of 1076 joints, 18 patients (1.7%) were reported to develop HO (table 4). McPherson 17 reported 3 cases HO associated with using large volumes of CS beads (average 33 cc). The majority did not require any surgical intervention and when the ossifications are symptomatic; a joint manipulation or removal at second stage revision were adequate. McPherson et al. 17 highlighted the possibility of scratching and damaging the articular surface of the artificial joint however they have not provided satisfactory supporting evidence.

### Reinfections

McPherson et al. 17 reported reinfections in 6 patients out 250 (2.4%) however, McPherson et al. series 17 included aseptic revision as well as revision for PJI. Flierl et al. 19 who included only DAIR procedures for hip and knee PJIs, reported poor outcomes with a 48% reinfection rate. They concluded that CS beads are not recommended for routine use in the management of PJI due to high cost and complications. Lum et al. 20 reported the highest success rate with no case of reinfection, however, only 14 joints out of 56 were revision due to PJI. Kallala 2015 18 reported only one case (6.7%) of reinfection out of 15 joints treated with revision for PJI. Kallala et al. 2018 paper 21 did not report the reinfection rates and hence there are 23 patients out 356 (6.5%) developed reinfection following the use of CS Beads.

## Discussion

According to this review, authors have reported variable results. With a 48% re-infection rate, Flierl et al. 19 concluded that the addition of antibiotic-loaded CS beads does not seem to improve outcomes of DAIR procedures in the setting of acute hematogenous or acute postoperative PJI. They suggested that 48% represents a best-case scenario as the follow-up period was very short and there may be more failures with a longer follow-up. Comparing these to Iza et al. results 23, who reported on 22 knees with PJI underwent DAIR only with an overall success rate of 77%, will make it unsatisfactory. However, Flierl et al. results are significantly better when compared to Uriarte et al. 24 who reported on 26 hips with PJI underwent DAIR only procedure with an overall success rate of 26.9%. A literature review by Tsang et al. 25, included 1296 patients who underwent DAIR for the management of PJI in hip arthroplasty, concluded that the only determinants of outcome is the timing of DAIR after the onset of symptoms of infection. Flierl's 19 paper did not explain the duration of symptoms, the time of diagnosis of PJI and the timing of the DAIR procedure which might have influenced the establishment of the biofilm and subsequent failure of DAIR procedure. It's our opinion that Flierl et al. results 19 might indicate that antibiotic-loaded CS beads are not effective in the presence of the biofilm. Kallala et al. 2015 18 reported reinfection in one joint out of 15 (6.7%) however, all patients in this cohort undergone revision surgery instead of DAIR. Kallala et al. 2015^18^ and 2018 21 papers were the only to report hypercalcaemia as a complication of using CS beads with an average incidence of 5.7%. The majority were asymptomatic and rarely required active treatment. Only one patient with hypercalcaemia needed admission to intensive care unit with successful recovery. Kallala et al. 2015^18^ and 2018^21^ recommended screening patients for hypercalcemia following implantation of CS beads.

3.8% had a persistent wound discharge which was managed conservatively in most of the cases and occasionally required surgical interventions. In a report by Menon et al. 26 of a case series of 39 cases with variable bone infection including osteomyelitis, non-union, and implant infection treated with surgical debridement and antibiotics loaded CS beads, they recommended careful interpretation of wound discharge as it can spontaneously subside without intervention in four to six weeks. They recommend monitoring other clinical signs of infection and blood analysis to avoid unnecessary re-operation. HO presented in 1.7% of joints. The effect of HO on patients' outcomes was not significant as majority were asymptomatic. Furthermore, it was reported to be brittle and easy to remove during second stage procedures.

We also noticed that Lum et al. 20 added Cefazolin to Vancomycin and Tobramycin and they had no cases of post-operative infection. On the other hand, the rest used only Vancomycin and Tobramycin, or Gentamycin and they reported variable re-infection rates in their series. The review has some limitations which include the level of evidence reviewed, the small number of studies and having most of the patients from one study. Only 2 papers have included only cases with PJI 18, 19. The other included papers 17,20,21 have variable case-mix including aseptic and septic revision with variable intervention ranging from DAIR to one and two stages revision. The largest number of cases in this review is coming from Kallala 2018^21^ paper with 755 joints. Only 387 out of Kallal's 755 were revision due to PJI. Kallal's 2018^21^ paper only reported CS beads complications and did not report the reinfection rate. In addition to the case-mix and the heterogenicity of the cases included in those selected papers, we are unable to draw a valid conclusion on the validity of antibiotics loaded CS bead in the management of PJI of hip and knee arthroplasty.

## Conclusion

The use of CS beads in the treatment of PJI is a useful technique in delivering high doses of antibiotics locally. CS beads can accommodate both heat and non-heat stable antibiotic as well as antifungal, in addition to its complete absorbability. Variable outcomes and success rates have been reported in the reviewed papers. A favourable outcome was reported when antibiotics loaded CS beads are used as an adjuvant to revision procedures compared to DAIR procedures. A small number of wound discharges, heterotrophic ossifications and hypercalcaemia cases were reported, and majority were asymptomatic. There has been an increase in complications when higher volumes of beads are used, especially in subcutaneous structures and in high-risk patients.

## Figures and Tables

**Table 1:** The result of the quality assessment.

Criteria/ Author	McPherson (17)	Kallala 2015 (18)	Flierl (19)	Lum (20)	Kallala 2018 (21)
1. Was the study question or objective clearly stated?	YES	YES	YES	YES	YES
2. Was the study population clearly and fully described, including a case definition?	YES	YES	YES	YES	YES
3. Were the cases consecutive?	YES	YES	YES	NO	YES
4. Were the subjects comparable?	NO	NO	NO	NO	NO
5. Was the intervention clearly described?	YES	YES	YES	YES	YES
6. Were the outcome measures clearly defined, valid, reliable, and implemented consistently across all study participants?	NO	YES	NO	YES	YES
7. Was the length of follow-up adequate?	YES	YES	YES	YES	YES
8. Were the statistical methods well-described?	NO	NO	YES	YES	YES
9. Were the results well-described?	YES	YES	YES	NO	YES
Quality Rating, ≥ 7 = Good, 5-6 = fair, ≤ 4 = poor	fair	good	good	fair	good

**Table 2:** Studies description.

Author/Year	Patients	Joint	Selection criteria	Joints due to PJI	follow-up protocol	Outcomes measures
McPherson 2013 (17)	250 joints	142 knees and 108 hips	aseptic revision of THR & TKR, two-stage septic revision and one stage DAIR for acute PJI.	126 joints (50%)	3 months, 6 months and a one-year postop.	reinfections and complications (instability, stiffness, extensor lag, HO, wound drainage, Kidney injury)
Kallala 2015 (18)	15 joints 8 males 7 females Mean age 64.8	6 knees and 9 hips	infected hip or knee arthroplasty.	15 joints (100%)	6 weeks, 3 and 6 months, 1 year	reinfections and complications (discharge, hypercalcaemia, and HO)
Flierl 2017 (19)	33 joints 22 males 11 females Mean age 62	6 hips and 27 knees	acute infection of hip or knee arthroplasty either postoperative or hematogenous. All underwent DAIR and antibiotics loaded CS beads.	33 joints (100%)	minimum of 3 months or until failure (3-30 months) mean 12.7 months	the primary outcome parameter was the recurrence of infection according to MSIS criteria
Lum 2018 (20)	56 joints	30 hip and 26 knees	as prophylaxis in high-risk patients undergone primary TKR or THR and revision TKR or THR with established infection (14 joints)	14 joints (25%)	2weeks, 6 weeks, 12 weeks and annually thereafter	primary outcomes included evaluation of wound complications, systemic toxicity, heterotrophic ossification, and need for further surgery. Secondary outcomes included reoperation and reinfection rates
Kallala 2018 (21)	755 joints 374 males 381 females Mean age 63	456 Knee and 299 Hip	all revisions due to infection (a majority of cases =387), DAIR, aseptic loosening, instability, peri-prosthetic fracture, metal allergy, implant failure, clinical need (pain and stiffness)	387 joints (51%)	6 weeks,3, 6, 12 months and bi-annually thereafter	complications of CS (wound drainage, hypercalcaemia, HO)

**Table 3:** The antibiotic beads.

Author/Year	Beads manufacturer	mixing procedure & antibiotics	Implantation technique
McPherson 2013 (17)	Stimulan, Biocomposites, Ltd., Keele, Uk	1 g of **Vancomycin** with each 10 cc of **CS** (20 g) and 240 mg of liquid **Tobramycin** (40 mg/ml) was added.	Knee: along the medial and lateral gutters of the knee, just before closure. Hip: the deep hip space inferior to the acetabulum and around the proximal femur. No beads were placed in the subcutaneous layer.
Kallala 2015 (18)	Stimulan, Biocomposites Ltd, Keele, UK	**CS** powder was mixed with 1 g of **Vancomycin** and 240 mg of **Gentamicin** per 10 cc of bead mixture with sterile water	implanted around the hip or knee joint, prosthesis, or spacer, before wound closure. No beads were placed subcutaneously or within adipose tissue.
Flierl 2017 (19)	Stimulan, Biocomposites INC, Wilmington, NC	10 cc **CS** with 1 g of **Vancomycin** and 1.2 g **Tobramycin**	placed into the wound before deep wound closure.
Lum 2018 (20)	Stimulan, Biocomposites INC, Wilmington, NC	**CS** with 1g **Vancomycin** powder, 1.2 g **Tobramycin** powder, and 1g **Cefazolin** powder	placed into the wound during final closure
R. Kallala 2018 (21)	Stimulan, Biocomposites Ltd, Keele, UK	1 g of **Vancomycin** with each 10 cc of CS (20 g) and 240 mg of liquid **Tobramycin** (40 mg/ml) was added. In patients with a fungal infection, 50 mg of **Amphotericin B** was also added.	implanted around the components or the spacer before the wound was closed

**Table 4:** Complications.

Author/Year	resorption/ weeks	Persistent drainage	Hypercalcaemia	Recurrence of infection	Heterotropic ossifications
McPherson 2013 (17)	average of 12 weeks	8 cases	not reported	6 cases (3 knees and 3 hips)	3 cases
Kallala 2015 (18)	a mean of 31 days (21 to 45)	no patient has wound discharge or dehiscence	3 patients	one patient	one patient
Flierl 2017 (19)	not reported	not reported	not reported	48% has failed, 16 of 33 patients at a mean of 13 months (3-30 months). 7 patients of those underwent 2 stage revision and 9 patients on chronic antibiotic suppression.	not reported
Lum 2018 (20)	6 weeks.	one case (1.7%) of persistent wound drainage occurred in a patient with type 2 diabetes mellitus and smoker whom revision TKA was performed	not reported	no post-operative infections were seen in any of the patients	one patient (1.7%)
R. Kallala 2018 (21)	not reported	32 patients	41 patients (5.4%), 22 knees and 19 hips	not reported	13 patients (1.7%), five knees and eight hips.
